# Microfluidic-mass spectrometry analysis of blood–brain barrier transport using engineered microparticle interfaces

**DOI:** 10.1039/d5sc10112c

**Published:** 2026-02-23

**Authors:** Shiyu Chen, Yingrui Zhang, Zengnan Wu, Tianze Xie, Tong Xu, Yi Zhang, Xianli Meng, Jin-Ming Lin

**Affiliations:** a School of Health and Rehabilitation, Chengdu University of Traditional Chinese Medicine Chengdu China; b Beijing Key Laboratory of Microanalytical Methods and Instrumentation, Key Laboratory of Bioorganic Phosphorus Chemistry & Chemical Biology (Ministry of Education), Department of Chemistry, Tsinghua University Beijing 100084 China wzn@mail.tsinghua.edu.cn jmlin@mail.tsinghua.edu.cn; c Chinese Medicine Germplasm Resources Innovation and Effective Uses Key Laboratory of Sichuan Province, Institute of High Altitude Multimorbidity/TCM-Integrated High Altitude Medicine Center, Hospital of Chengdu University of Traditional Chinese Medicine Chengdu China xlm999@cdutcm.edu.cn; d School of Ethnic Medicine, Chengdu University of Traditional Chinese Medicine Chengdu 611137 China

## Abstract

The blood–brain barrier (BBB) is vital for maintaining central nervous system (CNS) homeostasis but represents a formidable obstacle to drug delivery, underscoring the need for physiologically relevant *in vitro* models for CNS drug screening. However, existing *in vitro* BBB models remain limited in their ability to simultaneously recapitulate multicellular architecture, spatial anisotropy, and dynamic transport behavior while enabling real-time, quantitative analysis. Here, we present an integrated microfluidic-mass spectrometry platform that utilizes engineered BBB particles (BBBps) as a functional analytical interface to enable dynamic, *in situ* monitoring of BBB transport and metabolism. Using a microfluidic-aerosol hybrid process, we generate multicompartmental microparticles featuring a Matrigel-modified rough surface that enhances endothelial adhesion and tight junction formation, while their cores co-encapsulate microglia, neurons, and astrocytes to establish controlled neurovascular interactions. The BBBps exhibit size- and lipophilicity-dependent permeability, hypoxia-responsive antioxidative protection, and barrier-limited metabolism of salidroside. Real-time analysis with a Chip-MS system further reveals attenuated efflux of glutamate and lactate, confirming barrier integrity and metabolic regulation. This layered design of the micro-blood–brain barrier, combined with Chip-MS analysis, provides a scalable and functionally faithful *in vitro* model platform for studying drug transport, metabolism, and neurovascular studies.

## Abstract 2

A barrier-mimetic analytical interface integrating multicellular blood–brain barrier particles with online metabolite detection enables dynamic interrogation of permeability-metabolism coupling for drug screening.

## Graphical abstract

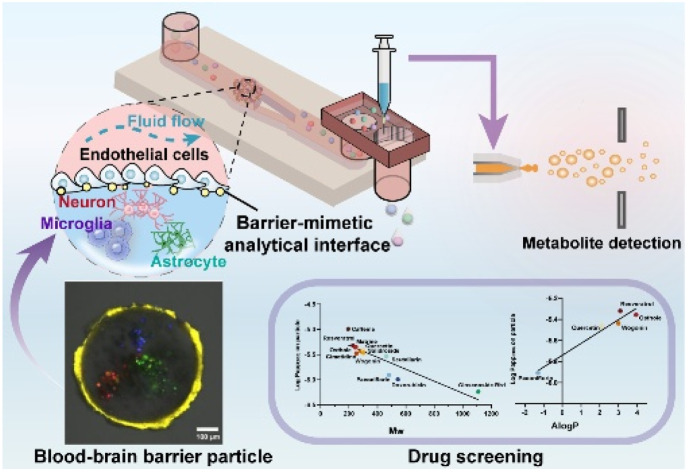

## Introduction

The blood–brain barrier (BBB), composed of brain microvascular endothelial cells, a basement membrane, and astrocytes, serves as a highly selective physiological barrier that maintains central nervous system (CNS) homeostasis by tightly regulating molecular transport between the blood and neural tissue.^1–3^ While this barrier protects the brain from neurotoxic insults, its tightly controlled transport complicates CNS drug development and mechanistic studies of neurovascular function. In this context, the BBB is not only a biological obstacle but also an analytically challenging system.^4–6^ Therefore, there is a pressing need for analytical platforms that can quantify transport and metabolism across BBB-like interfaces dynamically, *in situ*, and with high sensitivity.

Conventional *in vitro* BBB models, such as transwell co-culture systems, have advanced the understanding of endothelial permeability and intercellular signaling.^7,8^ However, their planar architecture and reliance on multi-layer assembly hinder spatial fidelity, throughput, and reproducibility, particularly when mimicking the three-dimensional (3D) neurovascular unit (NVU). Although microfabrication approaches including soft lithography and 3D bioprinting have improved structural complexity, they often require complex fabrication workflows and remain challenging to scale or couple seamlessly with continuous detection methods such as mass spectrometry (MS).^9–13^

In contrast, microfluidic droplet-derived hydrogel microparticles offer advantages such as uniformity, customizability, monodispersity, and high throughput.^14,15^ These microparticles exhibit excellent extracellular matrix (ECM)-mimicking properties, enabling effective multi-component cell co-culture and establishing them as ideal cellular carriers.^16^ Their monodispersity, tunable composition, and high-throughput production enable controlled mass transport and reproducible microenvironments, while their extracellular-matrix (ECM)-mimicking properties support multicellular co-culture. Diverse architectures, such as core–shell,^17,18^ non-spherical,^19,20^ and multicompartmental particles,^21^ have been explored for tissue modeling and cell delivery. Nevertheless, most existing microparticle systems lack the anisotropic architecture and defined cellular organization required to recapitulate BBB barrier functions, limiting their use for spatially resolved chemical analysis.^22^ An effective analytical strategy would thus require (i) precisely engineered physical boundaries for compartmentalized organization of BBB-relevant cell populations, (ii) anisotropic interfacial microenvironments that guide heterotypic cellular responses and regulate diffusion pathways, and (iii) modular architectures that are inherently compatible with online analysis to enable continuous monitoring of barrier transport and metabolism.

**Fig. 1:**
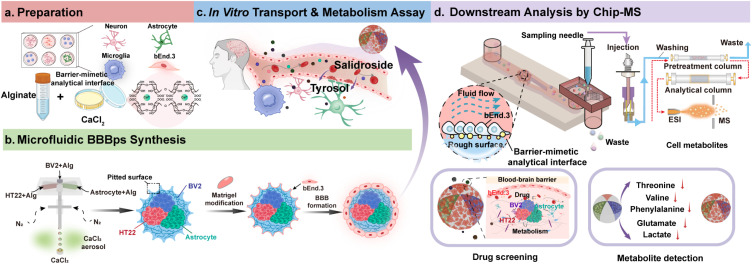
A hydrogel microparticle engineered with hierarchical tissue architecture and BBB functionality to simulate the neurovascular unit *in vivo*. (a) Preparation of cells and reagents. (b) Schematic diagram of the BBBps construction process. (c) Schematic diagram of the scale of the neurovascular unit in the brain. (d) Schematic diagram of drug screening and metabolism across the BBB, including the Chip-MS online detection system and endogenous metabolism and drug metabolism of BBB-related cells.

Here, we introduce an integrated fabrication-to-analysis workflow that couples microfluidic–aerosol-engineered BBB hydrogel microparticles (BBBps) with Chip-MS, establishing a multicellular barrier-mimetic analytical interface for real-time interrogation of BBB permeability and metabolism (Fig. 1). The fabrication process yields anisotropic, multicompartmental microparticles with a Matrigel-modified rough surface that supports endothelial adhesion and enhances tight-junction formation, while the inner compartments co-encapsulate neurons, astrocytes, and microglia to establish metabolically active NVU-like crosstalk. The multicompartmental 3D architecture provides controlled spatial positioning and proximity among encapsulated cell types while allowing intercompartment exchange of paracrine factors and metabolites, thereby supporting localized NVU-like interactions. In contrast to conventional microfluidic BBB models that rely on planar, membrane-based architectures and predominantly end-point sampling, the proposed BBB interface is directly compatible with microfluidic handling and online MS coupling, enabling continuous, time-resolved chemical measurement of transport and metabolism under controlled flow. Using this platform, we quantified *trans*-barrier transport for twelve compounds spanning a range of physicochemical properties, characterized salidroside metabolism, and monitored endogenous metabolite fluxes *via* real-time Chip-MS coupling. Collectively, this work introduces a microparticle-enabled analytical interface that links multicellular barrier architecture with continuous MS detection, providing a versatile approach for dynamic analysis and compound screening in BBB-like systems.

## Results and discussion

### Generation of multicompartmental hydrogel microparticles

The lab-made microfluidic system for the generation of multicompartmental microparticles includes a microfluidic chip for microparticle layout customization, gas-shearing coaxial needles for particle breakup, and an aerosol ejector for microparticle surface processing (Fig. S1). During the experiment, a 2% sodium alginate solution was used to prepare the multicompartmental microparticles. The chip features six asymmetrically arranged microchannels, allowing spatially controlled introduction of sodium alginate mixed with three different cell types (Fig. S1a). The central part of the chip connects to the inner needle of the droplet generator, while the nitrogen outlet is linked to the space between the inner needle and outer shell (Fig. S1b). Nascent multicompartmental alginate droplets were formed under nitrogen shear force. These droplets then passed through a CaCl_2_ atmosphere produced by the aerosol ejectors, where their surfaces were impacted by ultra-fine aerosol sprays. This process induced an uneven aggregation of calcium alginate, freezing the transient topological morphology and preventing the contraction and disappearance of surface structures due to the gravitational potential energy from fluid displacement. The droplets were then fully solidified in a downstream collection bath, resulting in rough-surfaced multicompartmental alginate microparticles, which were further modified by soaking in 5% Matrigel.

Under microscopic observation, the untreated microparticles have a rough surface texture (Fig. S2a). The coefficient of variation (CV) of size was under 5%, indicating good monodispersity, which is favorable for uniform cell assembly (Fig. S2b). Furthermore, the stability of the multicompartmental microparticles was assessed by incorporating fluorescent nanoparticles of different colors into the sodium alginate solution. Fluorescence intensity analysis indicated that the fluorescence occupancy of each compartment was similar, confirming clear segregation between particle regions (Fig. S2c).

### Construction of BBB microparticles

**Fig. 2:**
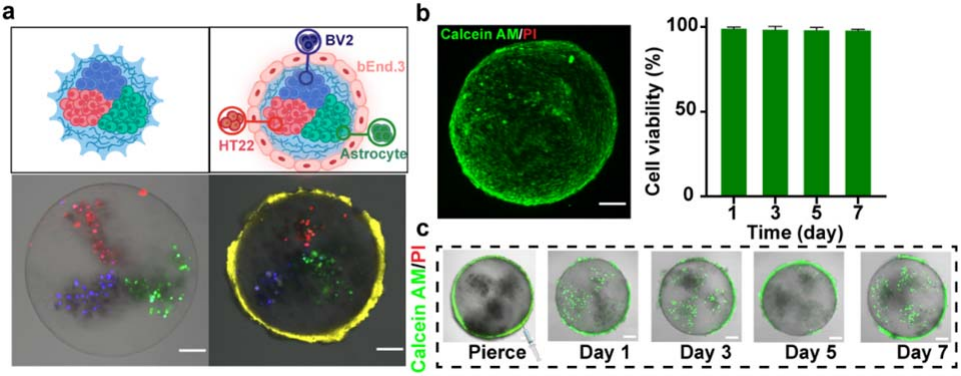
The preparation of BBBps. (a) Cell tracker characterization of internal compartments and external cell adhesion on BBBps. (b) The 3D layer scan image of BBBps; the surface barrier maintains high activity within 7 days (*n* = 4). (c) External and internal cells in BBBps after 7 days maintained high viability. The dense barrier structure formed by bEnd.3 on the surface of microparticles hindered the entry of fluorescent dyes, which was pierced to allow staining of internal cells. Scale bars = 100 µm.

The above-mentioned multicompartmental microparticles were loaded with three BBB-relevant types (BV2 microglia, HT22 neurons, and astrocytes), each confined to a separate compartment to facilitate downstream tracking and analysis of individual cell populations, while bEnd.3 endothelial cells were cultured on the particle surface using the hanging drop method.^23^ Although alginate microparticles are generally considered biopolymers with low cell adhesion properties, their rough surface structure and matrix modification provided a supportive microenvironment that promoted cell attachment.^24,25^ Previous research has shown that materials with surface roughness and chemical modifications can promote cell adhesion through interactions with cell membrane receptors and proteins.^24^ As a result, we observe that cells were firmly attached to the particle surfaces (Fig. 2a).

To assess the functionality of the BBBps, we evaluated the viability and function of the loaded cells. Cell growth and morphology were monitored using the Calcein-AM/EthD-1 double-staining kit. On the third day of culture, 3D imaging showed that the cells had spread across the microparticle surface, forming a uniform and complete barrier while maintaining high growth activity for 7 days (Fig. 2b). Interestingly, during the culture process, we observed that this barrier layer hinders the entry of small molecule substances such as fluorescent reagents. Therefore, in order to observe the viability of cells inside the microparticles, we performed precise microperforation using a syringe needle, allowing fluorescent reagents to penetrate. Over a course of 7 days, a large number of cells were observed to proliferate, while the cells both inside and on the surface of the microparticles maintained high viability (>98%) (Fig. 2c).

### Function of BBB microparticles

**Fig. 3:**
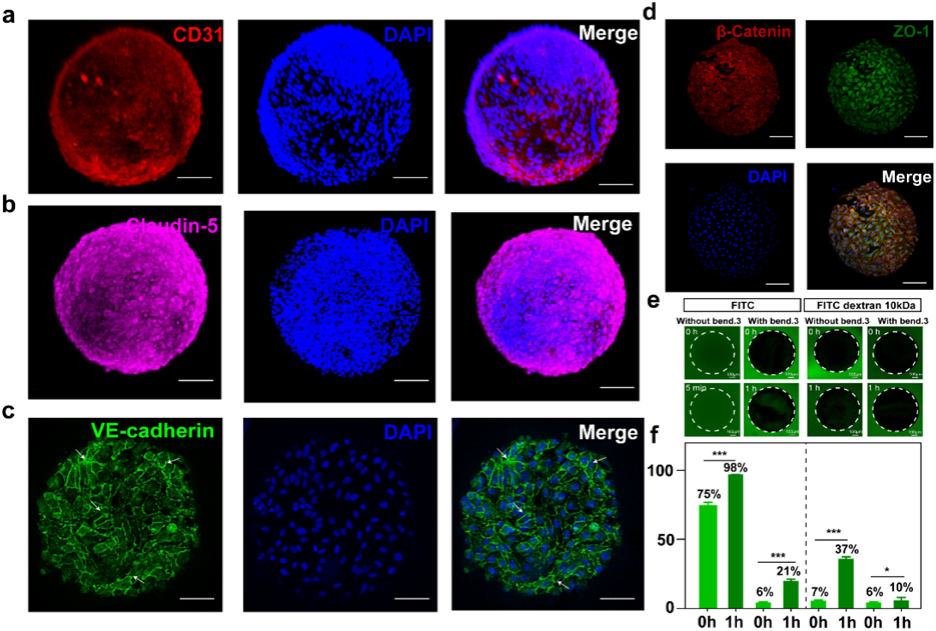
Fluorescence images of proteins related to barrier formation on the surface of BBBps and evaluating the barrier function by the permeability of BBBps and non-BBBps (microspheres without bEnd.3 on the surface). (a) CD31. (b) Claudin-5. (c) VE-cadherin. (d) Dual staining images of β-catenin and ZO-1. (e) Fluorescence imaging of FITC and 10 kDa FITC-dextran entering the internal space through BBB-like microspheres. (f) The penetration efficiency of non-BBBps and BBBps. The penetration efficiency is the ratio of the fluorescence intensity of tracers inside particles to the surroundings. *p < 0.05, **p < 0.01, ***p < 0.01, one-way ANOVA with Tukey's multiple comparison test. Data are presented as mean values ± SD. Scale bars = 100 µm.

To further verify the feasibility of the constructed BBBps, immunofluorescence staining was employed to confirm the formation of tight junctions between adjacent cells, validating the creation of the BBB. After 3 days of culture, CD31 (a marker of endothelial cells,^26^ indicating vascularized-like expression) (Fig. 3a) and Claudin-5 (a key tight junction protein in the BBB)^27^ (Fig. 3b) were both highly expressed, demonstrating good vascularized-like characteristics and tight junction formation. VE-cadherin (a key component of intercellular adhesion junctions) staining showed a continuous filamentous pattern, which further confirmed that endothelial cells formed a continuous monolayer on the surface (Fig. 3c).^28^ Double staining for β-catenin (essential for regulating and maintaining the barrier by connecting cadherins to the actin cytoskeleton)^29^ and ZO-1 (contributing to barrier integrity and controlling intercellular permeability)^30^ further supported the integrity of the barrier and the maintenance of tissue homeostasis (Fig. 3d). The tight junctions between brain vascular endothelial cells restrict the entry of hydrophilic macromolecules into the brain. Thus, the selective permeability of hydrophilic non-ionic tracers is commonly used to assess the integrity of tight junction formation between endothelial cells.^31^ Here, both BBBps and non-BBB microparticles (non-BBBps) were exposed to solutions containing fluorescein isothiocyanate (FITC) and FITC-labeled dextran of 10 kDa (Fig. 3e). The fluorescence distribution inside and around the particles was measured to assess barrier function. In the non-BBBps group, FITC diffusion reaches equilibrium rapidly within 5 min, resulting in similar fluorescence intensities inside and outside the particles. In contrast, the BBBps significantly inhibited FITC diffusion, with remarkably reduced diffusion even after 1 h. This trend was consistently observed with another tracer, confirming the successful establishment of selective permeability in BBBps (Fig. 3f).

### Evaluation of BBB protective function in BBBps

**Fig. 4:**
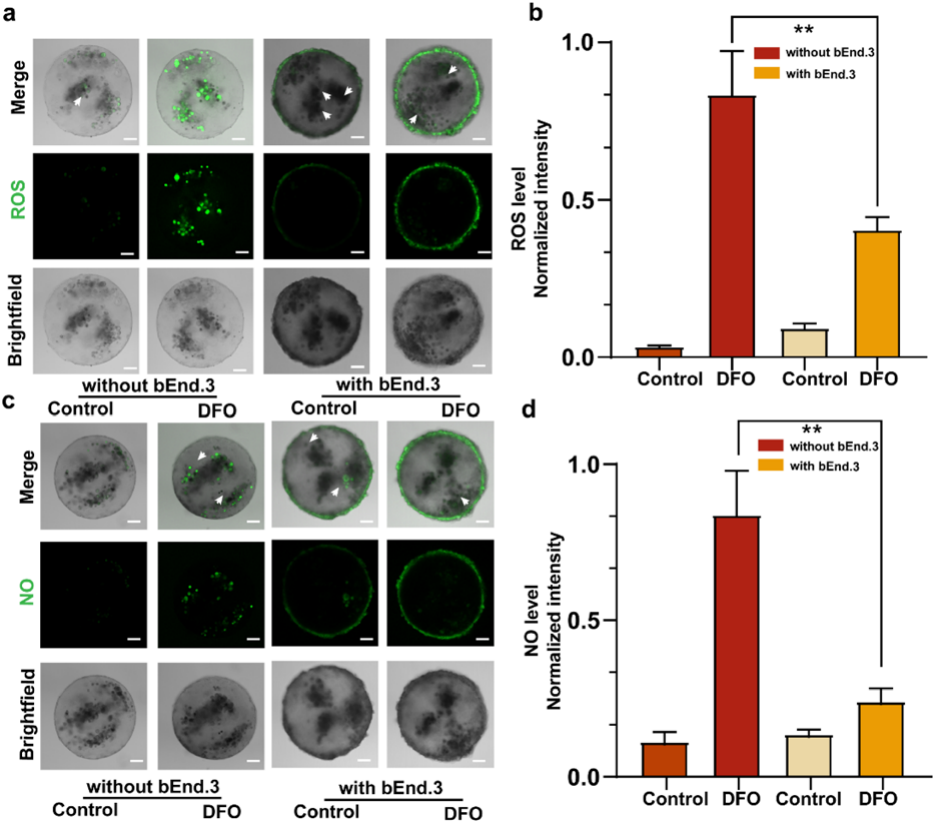
Permeability of active ingredients of different molecular weights on BBBps. Evaluation of the BBB protective function of BBBps against ROS and NO. (a and b) The ROS level induced by DFO in non-BBBps and BBBps (*n* = 5). (c and d) The NO level induced by DFO in non-BBBps and BBBps (*n* = 5). *p < 0.05, **p < 0.01, ***p < 0.01, one-way ANOVA with Tukey's multiple comparison test. Data are presented as mean values ± SD. Scale bars = 100 µm.

The BBB selectively permits the passage of specific substances to protect the brain from toxins and large molecules. Factors such as oxidative stress and neuroinflammation can lead to BBB damage.^32,33^ Nitric oxide (NO) and reactive oxygen species (ROS) are key signaling molecules involved in the body's hypoxic response,^34,35^ and their excessive accumulation can result in acute brain damage and impaired BBB function.^36^ The production levels of NO and ROS were measured to evaluate BBB damage under hypoxic conditions induced by deferoxamine (DFO).^37,38^ After 12 hours of DFO exposure, NO and ROS levels inside BBBps and non-BBBps were assessed using DCFH-DA and DAF-FM fluorescent probes. The results showed that BBBps exhibited significantly suppressed production levels of NO (Fig. 4a and b) and ROS (Fig. 4c and d) compared to non-BBBps, demonstrating the protective effect of the BBB structure. These findings demonstrate that BBBps successfully mimicked the structural and functional characteristics of the BBB on the single-particle level, exhibiting protective capabilities under hypoxic stimulation.

### Assessment of drug permeability across the BBB using BBBps

**Fig. 5:**
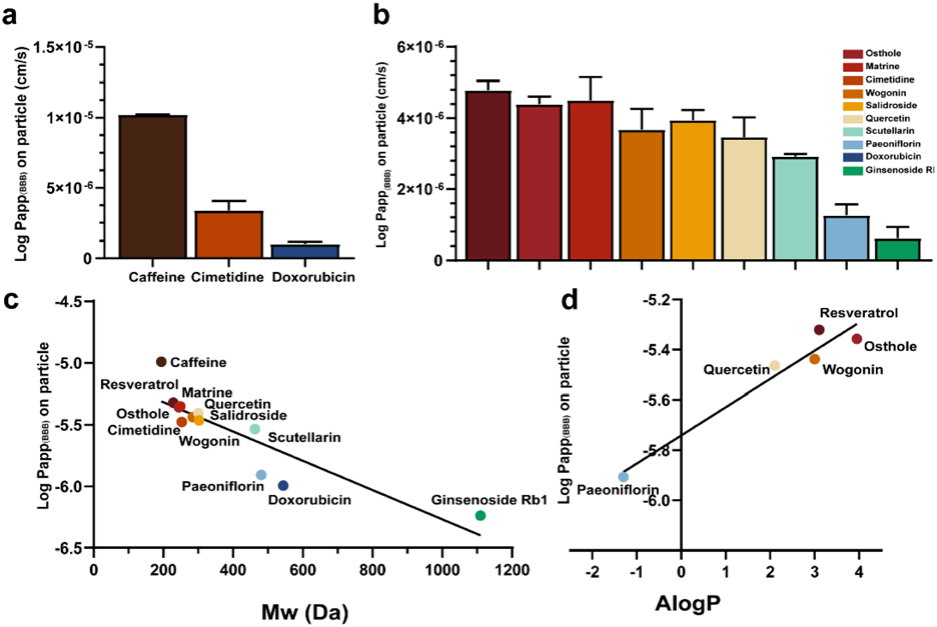
Permeability of active ingredients of different molecular weights on BBBps. (a) Permeability of caffeine, cimetidine, and doxorubicin in the BBBps model. (b) Permeability of 12 active substances in the BBBps model. (c) Linear relationship between the molecular weight and permeability of 12 active substances in the BBBps model (*R*^2^ = 0.7433). (d) Relationship between the octanol/water partition coefficients and *Papp* (BBB) of resveratrol, osthole, wogonin, quercetin, and paeoniflorin (*R*^2^ = 0.8903).

Next, we tested the permeability of three small molecule drugs on the BBBps model: (1) caffeine, a lipophilic small molecule that can rapidly permeate the BBB through simple diffusion and carrier-mediated transport; (2) cimetidine, a moderately permeable molecule transported by efflux transporters and organic cation transporters; (3) doxorubicin, known for its poor BBB penetration. ESI-MS was used to quantify the drugs, detecting caffeine (*m*/*z* 194.1 → 138.2), cimetidine (*m*/*z* 253.0 → 159.1), and doxorubicin (*m*/*z* 543.9 → 361.2) (Table S1). Calibration curves were constructed by spiking different concentrations of each drug (Fig. S3). Compared with the *in vivo in situ* perfusion data reported in the literature, these results in BBBps closely resemble the *in vivo* situations (Table S2),^39,40^ indicating the formation of a functional high-fidelity BBB unit in the BBBps model.^41^ This suggests its suitability for subsequent experiments on drug permeability across the BBB (Fig. 5a). Subsequently, nine active ingredients from traditional Chinese medicine of different molecular weights were further tested, including resveratrol (*m*/*z* 227.1 → 143.2), osthole (*m*/*z* 244.9 → 131.2), matrine (*m*/*z* 249.1 → 148.3), wogonin (*m*/*z* 284.8 → 270.1), salidroside (*m*/*z* 299.0 → 119.0), quercetin (*m*/*z* 302.8 → 165.2), scutellarin (*m*/*z* 461.0 → 285.0), paeoniflorin (*m*/*z* 525.0 → 121.2), and ginsenoside Rb1 (*m*/*z* 1107.4 → 783.5) (Table S1). The permeability coefficients for each drug showed that substance permeation through the BBB was closely related to molecular weight, with larger molecules facing greater difficulty in crossing the BBB. Additionally, the physical and chemical properties of compounds influenced their BBB permeability (Fig. 5b and c and Table S3). Lastly, we investigated the relationship between the octanol/water partition coefficient (*K*_ow_) and the permeability of five active ingredients (Paeoniflorin, Quercetin, Wogonin, Resveratrol, and Osthole) across the BBB (Fig. 5d). Studies confirmed that the BBB, as a highly selective and dynamic lipophilic barrier, favors substances with higher *K*_ow_ values, indicating that greater lipid solubility facilitates BBB penetration.

### Quantification of salidroside and tyrosol on the BBBps

**Fig. 6:**
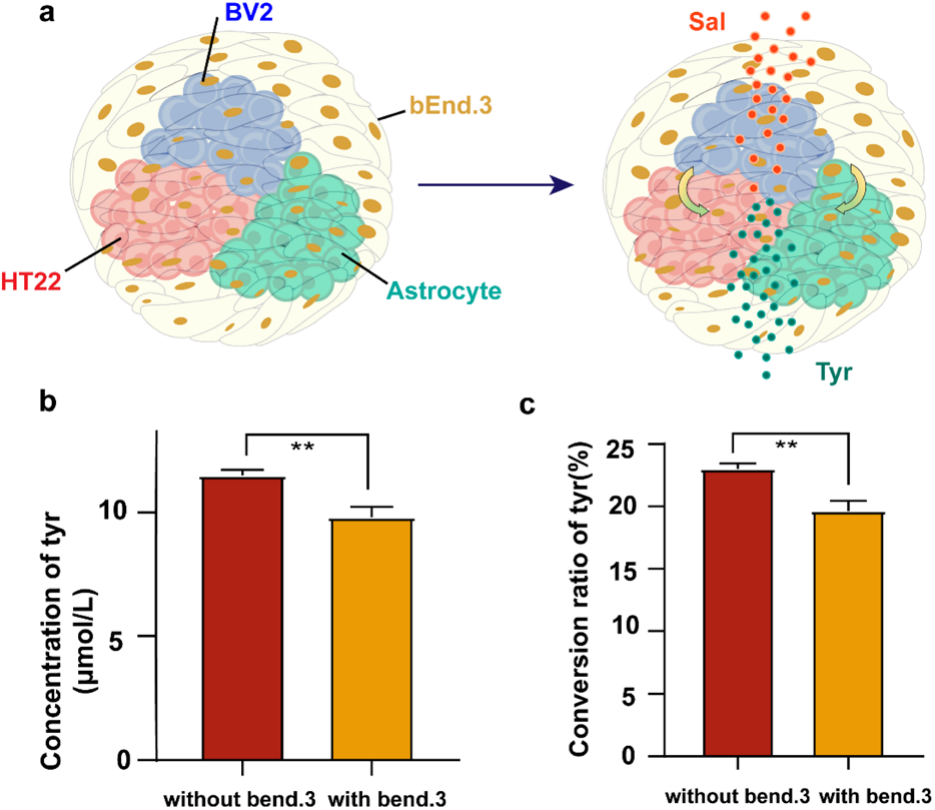
Assessment of the conversion of salidroside to the drug-reactive metabolite tyrosol on BBBps. (a) Schematic of salidroside transformation in the BBBps. (b) The yield of the metabolic product tyrosol in cell culture medium after the non-BBBps and BBBps were treated with salidroside for 24 h. (c) Calculated percentage conversion of salidroside to tyrosol. *p < 0.05, **p < 0.01, ***p < 0.01, one-way ANOVA with Tukey's multiple comparison test. Data are presented as mean values ± SD.

To evaluate the functionality of the BBBps constructs, we assessed the metabolic activation of salidroside, the primary component of the Tibetan medicine *Rhodiola rosea* rhizoma et radix. Salidroside is known to mitigate hypoxia-induced metabolic disturbances and neuronal damage by preserving mitochondrial function.^42,43^ Its metabolite, tyrosol, also exhibits anti-hypoxic and anti-inflammatory properties. After crossing the barrier to reach the internal structure, salidroside began to metabolize into tyrosol (Fig. 6a). After treating BBBps with 50 µM salidroside for 24 hours, identification and quantification of salidroside and tyrosol were conducted using negative mode MRM (Table S4). A standard curve was drawn by preparing samples with different concentration gradients using the standard substance, and the concentrations of salidroside and tyrosol in the samples were calculated (Fig. S4). Quantitative analysis revealed that BBBps exhibited significantly lower levels of tyrosol (Fig. 6b) and a reduced metabolic conversion rate of salidroside compared to non-BBBps (Fig. 6c). These results further demonstrate that the BBBps model successfully simulates the *in vivo* phenomenon of diminished drug metabolic activity due to BBB interference.

### Effects of the BBB on amino acid metabolism in BBBps

**Fig. 7:**
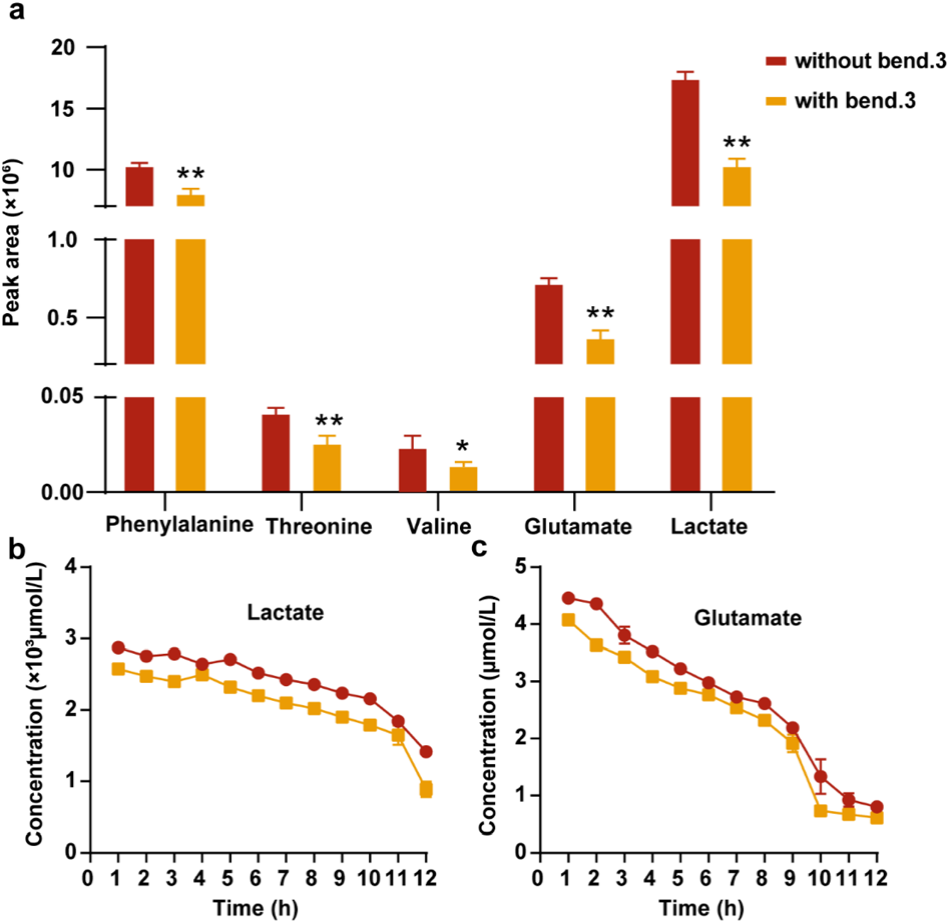
Dynamic monitoring of amino acid and lactate metabolites based on Chip-MS between non-BBBps and BBBps. (a) Differences in the changes of the contents of BBB marker amino acid and lactate metabolites. (b) Real-time quantitative analysis of lactate over 12 h. (c) Real-time quantitative analysis of glutamate over 12 h. *p < 0.05, **p < 0.01, ***p < 0.01, one-way ANOVA with Tukey's multiple comparison test. Data are presented as mean values ± SD.

We evaluated the impact of the barrier on the levels of specific intracellular endogenous metabolites, including amino acids and lactate, which serve as indicators of BBB functionality. The evaluation was performed by comparative analysis of secreted metabolite concentrations between BBBps and non-BBBps. Emerging evidence indicates that metabolites such as threonine, valine, phenylalanine, glutamate and lactate are critically involved in the modulation of BBB function.^44–46^ Among them, threonine, valine, and phenylalanine need to cross the BBB. When their concentrations are excessively elevated, they competitively inhibit the transport of other amino acids, leading to cerebral amino acid imbalance that disrupts neurotransmitter synthesis and protein metabolism. Notably, the BBB regulates intracerebral concentrations of threonine, valine, phenylalanine, glutamate, and lactate through specialized transporters and selective permeability mechanisms, thereby modulating their metabolic pathways and neurological functions.^47,48^ In our study, after 12-hour cultivation of BBBps and non-BBBps models, culture supernatants were collected to quantify five intracellular endogenous metabolites: threonine, valine, phenylalanine, glutamate, and lactate. The results demonstrated that threonine, phenylalanine, glutamate and lactate exhibited elevated efflux under compromised barrier integrity, attributable to increased BBB permeability (Fig. 7a). The intact BBB leads to a decrease in the efflux of these metabolites, with the reduction exceeding 20%. To further validate these findings, we dynamically monitored the 12 h concentration profiles of glutamate and lactate using a Chip-MS system for real-time metabolic flux analysis. The microfluidic chip design and internal instrument of Chip-MS are shown in Fig. S5. Fragmentation ions and patterns of glutamate and lactate were analyzed using reference standards in product ion scanning mode, with representative spectra shown in Fig. S5a and b. Detailed analytical parameters are provided in Table S5. Subsequently, calibration curves were established for reliable quantification of both metabolites (Fig. S5c and d), enabling hourly monitoring of their concentrations in real-time throughout the 12-hour dynamic cultivation process. The results demonstrated that compared to the non-BBBps group, our BBBps model attenuated the glutamate and lactate accumulation caused by increased BBB permeability, effectively suppressing their efflux to maintain cerebral metabolic homeostasis (Fig. 7b and c). These findings align with trends reported in prior literature.^44,45,47^ Overall, our results confirm that we have successfully established an *in vitro* BBB model on single particles and simulated the effects of the BBB on endogenous metabolites.

## Conclusions

In this study, we established an integrated BBBps-Chip-MS analytical platform in which microfluidic-engineered hydrogel microparticles serve as standardized multicellular barrier interfaces for BBB interrogation. This particle-based design enables spatially defined co-culture of brain-relevant cell types, programmable surface modification to support endothelial adhesion and tight-junction formation, and coupling with online mass spectrometry for dynamic and quantitative chemical analysis. The resulting BBBps exhibited key barrier phenotypes, including tight-junction protein expression and selective molecular permeability, and maintained barrier responsiveness under hypoxic stress. Leveraging continuous Chip-MS coupling, we quantified drug transport across the interface and observed permeability trends governed by molecular size and lipophilicity that are consistent with *in vivo* behavior. Importantly, the platform enabled dynamic, *in situ* monitoring of endogenous metabolite exchange, revealing markedly reduced efflux of neuroactive metabolites (*e.g.*, glutamate and lactate) under intact barrier conditions. Moreover, real-time metabolic profiling captured salidroside conversion and its dependence on barrier integrity, demonstrating the capability to resolve coupled transport-metabolism processes within a controlled microenvironment. Collectively, this work introduces a scalable and versatile analytical strategy that couples engineered multicellular barrier interfaces with online mass spectrometry, offering broad potential for quantitative CNS permeability assessment, metabolism studies, and barrier-associated phenotyping.

Looking forward, the platform can be further improved by enhancing batch-to-batch consistency in BBBp production, increasing the number of parallel channels for higher screening capacity, and strengthening multi-channel operational robustness for synchronized dosing, perfusion, and data acquisition. These advances will further improve scalability and reliability and broaden the utility of the BBBps-Chip-MS strategy for compound profiling and CNS-relevant phenotyping.

## Author contributions

Z. W., Y. Z., X. M. and J.-M. L. obtained the funds and supervised the project; S. C., Z. W. and J.-M. L. conceived the idea; S. C., Y. Z., and Z. W. designed the experiments; S. C. and Y. Z. performed experiments; S. C., Y. Z. and T. X. analyzed the data; and S. C. wrote the original manuscript. All authors revised and approved the manuscript.

## Conflicts of interest

There are no conflicts to declare.

## Supplementary Material

SC-017-D5SC10112C-s001

## Data Availability

The data supporting this article have been included as part of the supplementary information (SI). Supplementary information: materials and methods; the homemade microfluidic system for generating multicompartment particles; characterization of rough-surfaced multicompartment hydrogel microspheres; chromatograms, calibration curves, and ion fragmentation spectra of active ingredients with different molecular weights; fragment spectra and calibration curves for salidroside and tyrosol; photographs and schematics of the Chip-MS device; calibration information for glutamate and lactate; MRM parameters for target compounds and metabolites; permeability comparisons of model drugs (caffeine, cimetidine, and doxorubicin) in BBBps, transwell, and *in vivo* models; molecular weights and *P*_app_ data for 12 active ingredients; and LC gradient elution conditions for the target compounds and metabolites. See DOI: https://doi.org/10.1039/d5sc10112c.
