# Supplementary material for: Microfluidic-mass spectrometry analysis of blood–brain barrier transport using engineered microparticle interfaces
Source: Chem Sci. 2026 Feb 23;17(13):6371–8. doi: 10.1039/d5sc10112c (PMC12951469; doi:10.1039/d5sc10112c)
Supplement: SC-017-D5SC10112C-s001 [file SC-017-D5SC10112C-s001.pdf]

Supplementary Information (SI) for Chemical Science.
This journal is © The Royal Society of Chemistry 2026

Supplementary Information

Microfluidic-Mass Spectrometry Analysis of Blood-Brain Barrier Transport Using Engineered Microparticle Interfaces

Shiyu Chen, [a,b] Yingrui Zhang, [b,d] Zengnan Wu, [*b] Tianze Xie, [b] Tong Xu, [d] Yi Zhang, [d] Xianli Meng, [*c] and Jin-Ming Lin[*b]

[a] School of Health Preservation and Rehabilitation, Chengdu University of Traditional Chinese Medicine, Chengdu, China

[b] Beijing Key Laboratory of Microanalytical Methods and Instrumentation, Key Laboratory of Bioorganic Phosphorus Chemistry & Chemical Biology (Ministry of Education), Department of Chemistry, Tsinghua University, Beijing, 100084, China.

[c] Chinese Medicine Germplasm Resources Innovation and Effective Uses Key Laboratory of Sichuan Province, Institute of High Altitude Multimorbidity/TCM-Integrated High Altitude Medicine Center, Hospital of Chengdu University of Traditional Chinese Medicine, Chengdu, China

[d] School of Ethnic Medicine, Chengdu University of Traditional Chinese Medicine, Chengdu, 611137, China

Correspondence should be addressed: Zengnan Wu: E-mail: wzn@mail.tsinghua.edu.cn
Xianli Meng: E-mail: xlm999@cdutcm.edu.cn
Jin-Ming Lin: E-mail: jmlin@mail.tsinghua.edu.cn

TABLE OF CONTENTS

1. Materials and Methods S3-S7

2. Data supplementation S8-S17

3. Figure S1. Homemade multicompartmental particle generation microfluidic system. S8

4. Figure S2. Characterization of multicompartmental hydrogel microspheres with rough surfaces.S9

5. Figure S3. Chromatograms, standard curves, and ion fragmentation spectra of active ingredients with different molecular weights. S10

6. Figure S4. Fragment spectra and calibration curves for salidroside and tyrosol S11

7. Figure S5. Photographs of the microfluidic chip-mass spectrometry (Chip-MS) device. S12

8. Figure S6. The characteristic fragment structure and standard curves of glutamate and lactate in standard substances. S13

9. Table S1. The MRM mode parameters of 12 target compounds S14

10. Table S2. Comparison of permeability of model drugs (caffeine, cimetidine, and doxorubicin) in BBBps and in situ cerebral perfusion in rodents. S15

11. Table S3. Molecular weights and Papp data for 12 active ingredients S16

12. Table S4. The MRM mode parameters of salidroside and tyrosol S17

13. Table S5. The MRM mode parameters of target metabolites S18

14. Table S6. Gradient elution conditions for 12 active pharmaceutical molecules S19

15. Table S7. Gradient elution conditions for the metabolism of salidroside S20

16. Table S8. Gradient elution conditions for amino acids such as threonine and lactate.............S21

Materials and Methods

Chemicals and Materials

Sodium alginate (2%) and FITC-conjugated dextran (10 kDa) were purchased from Sigma-Aldrich (Missouri, USA). Matrigel was purchased from Corning (USA). Sterile calcium chloride, 0.9% normal saline, Dulbeccos modified Eagles medium (DMEM, with penicillin-streptomycin), trypsin, phosphate buffered solution (PBS), fluorescein isothiocyanate (FITC) were obtained from Solarbio (Beijing, China). Fetal bovine serum (FBS) was purchased from ExCell Bio (Shanghai, China). DAPI staining solution was purchased from Beyotime (Shanghai, China). Live/dead assay kit (Calcein AM/EthD-1), CD31 monoclonal antibody and the secondary antibody labeled with Alexa Fluor 555, 488 and 633 were obtained from Thermo Fisher Scientific (Shanghai, China). ZO-1 monoclonal antibody was purchased from Boster Biological Technology (Wuhan, China). Claudin-5 monoclonal antibody was purchased from ABclonal Technology (Wuhan, China). VE-cadherin Alexa Fluor 488 was purchased from Santa Cruz Biotechnology. SU-8 2050 negative photoresist and developer were purchased from Microchem Corporation (Newtown, MA). Isopropanol and 11H,1H,2H,2H-Perfluorooctyl trichlorosilane were obtained from Sigma-Aldrich (St. Louis, MO). Sylgard 184 poly(dimethylsiloxane) (PDMS) and initiators were purchased from Dow Corning (Midland, MI). Stainless steel coaxial needles and aerosol ejectors were purchased from the Taobao website (China). Water was purified by a Milli-Q ultrapure water system from Millipore.

Cell culture

BV2 microglia cells, HT22 hippocampal neuron, astrocytes, and brain-derived endothelial cells.3 (bEnd.3) were obtained from Cancer Institute & Hospital Chinese Academy of Medical Science. The four kinds of cells were cultured in the complete DMEM medium containing 10% FBS. The cells were maintained in a humidified atmosphere of 5% CO2 at 37 °C.

Fabrication of the microfluidic device

The microfluidic assembly system is composed of the microfluidic chip and a gas-shearing droplet generator, two aerosol ejectors, and a collection bath. The microfluidic chip was prepared using soft lithography. The SU-82050 photoresist was uniformly spun onto the 75 mm silicon wafer at a speed of 1250 rpm for 90 s in a spincoater (KW-4A, Microelectronics Center, Chinese Academy of Sciences) to a thickness of 140 μm. After soft baking at 70 °C for 5 min, the silicon wafer was patterned by applying UV exposure using a transparency photo mask. Then, the obtained pattern was rebaked at 75 °C for 20 min and developed with an SU-8 developer. After developing the microstructure, the mold was treated

with a silylation reagent (1H,1H,2H,2H-perfluorooctyl trichlorosilane) under vacuum conditions for 6h. Later on, the degassed 10:1 mixture of PDMS and initiators was poured onto the mold and cured at 65 °C for 3 h. Once the cured device was prepared, the PDMS stamp was peeled off from the mold pattern and the channel inlets and outlet were made by using the precision punch. The PDMS replicas were tightly bonded to the glass substrate after surface treatment by oxygen plasma (PDC-32G, Harrick Plasma, USA). A commercialized coaxial needle device was used as the gas-shearing droplet generator. The device was mainly composed of an inner needle (inner diameter: 220 μm, outer diameter: 400 μm) and an outer needle (inner diameter: 1050 μm, outer diameter: 1500 μm) which were designed coaxially. The inner needle of the coaxial needle is integrated with the prepared microfluidic chip and fixed vertically. A 1 mL injection syringe containing 2% w/v alginate solution mixed with cell suspension is connected to the microfluidic chip and the outer needle is connected to the nitrogen tank. Two ends of the $CaCl_2$ aerosol injector are symmetrically placed on the underside of the device. The vertical height (H_1) from the nozzle of the $CaCl_2$ aerosol sprayer to the tip of the coaxial needle is 8 cm. The collection vessel containing the $CaCl_2$ solution is placed on the ground perpendicular to the device. The height (H_2) from the center of the collection vessel to the tip of the coaxial needle is 11 cm.

Generation of Multicompartment Microparticles with a Rough Surface

A six-channel integrated microfluidic device was used to generate corresponding six-compartmental microspheres. Six 1 mL syringes (BD) filled with 2% w/v alginate solution (or alginate solution containing cells) were connected to the microfluidic chip channel with an inner needle integrated into the channel in the center of the chip. The two aerosol injectors are placed horizontally, and the collection bath containing 100 mM $CaCl_2$ solution is placed vertically on a flat surface. The alginate solution and nitrogen gas were injected into the inner channel and outer annular channel of the droplet generation system respectively. At the same time, the $CaCl_2$ aerosol injector immediately started to generate $CaCl_2$ mist, and the generated alginate droplets weaved in and out of the $CaCl_2$ mist and finally fell into the collection tank. After thorough cross-linking, the multicompartment rough microparticles were transferred and stored in the complete DMEM medium containing 10% FBS and 5 mM $CaCl_2$. In this experiment, the nitrogen flow was controlled by a rotameter at 1.4 L/min, and the flow rate of the alginate solution was controlled by a pump (Harvard) at 2500 μL/h. The diameter of the microspheres decreases as the gas flow rate increases[1].

Fabrication of a BBB in Multicompartment Rough Microparticles

BV2 cells, HT22 cells and astrocytes were mixed with 2% w/v alginate solution and added into a 1mL injection syringe and passed into a six-channel microfluidic chip connected to the internal needle of a coaxial needle. The preparation steps and conditions are the same as above. The generated multicompartment rough microparticles were then transferred to a solution containing matrigel for

surface modification, and then the bEnd.3 cell suspension (1.5×10^7 cells/ mL) was attached to the surface of the multicompartment rough microparticles for culture and incubation for 1 hour. After the cells adhered to the surface of the microparticles, fresh medium was added and excess cell debris was removed.

BBB Functionality Assay

To quantify and visualize BBB function by determining the penetration of fluorescent tracers into BBBps, the solution containing FITC and 10 kDa FITC-conjugated dextran was prepared at 25 μg/mL in the DMEM. These solutions were then added to microparticles with and without bEnd.3 monolayer and fluorescence images were taken at 0 h (immediately after injection) and 1 h later to evaluate by analyzing the diffusion of the FITC probe in the hydrogel microparticles. The penetration efficiency = FI_{inside} / $FI_{outside}$. FI_{inside} is the fluorescence intensity of fluorescent molecules diffused inside the microparticle, and $FI_{outside}$ is the fluorescence intensity outside the microparticle.

In addition, referring to the work of Shi[2] and Wang[3], HBSS was used to prepare the drug penetration working solution of caffeine, cimetidine, and doxorubicin with a concentration of 50 μM. Then equal amounts of microparticles loaded with bEnd.3 on the surfaces were added to the prepared solutions. Sample solutions were collected at 0 h (immediately after injection) and 1 h later. The collected sample solutions were subjected to HPLC quantitative analysis after pretreatment, and the apparent permeability coefficient (*Papp*) of the drug was calculated according to the following formula:

$$P_{app} = \frac{C_{(t)}V}{C_o A \Delta T}$$

In this context, Papp represents the apparent permeability coefficient, $C_{(t)}$ denotes the drug concentration permeating through BBBps, V stands for the volume of multicompartment particles, C_0 is the initial concentration of the drug, A represents the surface area of multicompartment microparticles and $_{\Delta}T$ is the analysis time.

Cell Staining and Analysis by Confocal Microscopy

For cell staining, Calcein AM/EthD-1 staining kit, Mitotracker Green (1 μM) Mitotracker Red (1 μM) and Mitotracker Blue (1 μM) and in phosphate buffer (PBS, 0.01 M, pH 7.4) was used to incubate the cell-laden microparticles at 37 °C for 20 min. After washing with normal saline, samples could be taken for imaging. In addition, the BBBps with and without protection of bEnd.3 brain endothelial cells and the production of ROS and NO after DFO induction were measured. After 72 h of culture, the culture medium was changed to a medium without FBS. After 24 h of DFO induction, ROS and NO probes were added. According to the manufacturer's instructions, the ROS and NO contents of the four groups were measured. In immunofluorescence, the cell-laden microparticles were washed with NaCl solution containing 100mM $CaCl_2$ and immersed in 4% paraformaldehyde for 20 min. The samples were then

permeabilized with 0.25% v/v Triton X-100 for 20min and then incubated with 5% (v/v) bovine serum albumin solution for 60 min at room temperature to block nonspecific binding. The samples were incubated with Vinculin primary antibodies at 4 °C overnight. Finally, the samples were stained with DAPI for 10 min at room temperature. All the fluorescence images were observed with an inverted confocal laser scanning microscope (Zeiss LSM 780).

Real-time Analysis of Metabolites in BBBps Using Chip-MS

The Cellent CM-MS device comprises an injection pump unit, a microparticles culture section, a changeover valve, and an LC-MS. The design of the chip is depicted in Fig. S6a. Circles with a diameter of 4 mm at both ends are utilized for mounting the collection pool. There are 6 channels, each having a width of 1 mm. The incubation chamber above the channels has a diameter of 2 mm, which can directly accommodate microparticles approximately 500 micrometers in size. The microchannels behind the culture chambers have a width of 200 μm and are inaccessible to particles, whereas the medium can circulate through them. According to the designed pattern, the polystyrene material with dimensions of 60×60 mm is machined to obtain a chip mold with a channel height of 800 μm. Then Polydimethylsiloxane is poured, and bonded to the glass after being dried to obtain the microfluidic chip. Chips containing 10 microparticles per culture chamber were inserted into the device. Referring to previous work[4], the microsyringes and pumps were used to inject the culture medium into the microchip; every microchannel would be incubated with a continuous flow of the culture medium at a rate of 20 μL/h. The homemade microchip loaded with particles was placed into the culture device. The syringes were filled with culture medium and connected to the microchannels with polytetrafluoroethylene tubes Collection pools fixed the outlet of microchannels for detecting metabolites (Fig. S6b). Use the automatic probe provided by the modified UPLC injector for accurate sampling, and the specific position and height can be programmed by software. In sampling mode, the automated probe transfers the culture medium from the collection reservoir through the filter to the analysis column, with excess liquid being drained through the waste outlet every hour. The metabolites collected within an hour are detected using the multiple reaction monitoring mode of a triple quadrupole mass spectrometer (Shimadzu 8050, Japan), while other analytes are flushed into the waste, enabling real-time dynamic detection (Fig. S6c). This direct coupling approach avoids sample loss, volatilization, and contamination associated with traditional offline collection, achieving minimized dead volume transfer from the cellular barrier model outlet to the detection endpoint. Details of the MS/MS transitions of each analyzed compound are provided in Supporting information (Table S1, S4-5). The elution mobile phase consisted of (A) UPW and (B) acetonitrile and was applied at a flow rate of 0.3 mL/min. The specific elution procedures for each substance are detailed in the Supplementary Information (Table S6-8). For triple quadrupole measurements (LC-QqQ-MS), the sample aliquots of 1 μL were injected into the chromatographic

column. A C_{18} column (100 mm × 2.1 mm, 3 μm; Shimadzu, Japan) was loaded on the liquid chromatography triple quadrupole tandem mass spectrometry (LC-QqQ-MS/MS, Shimadzu, Japan). The mass spectrometric instrument parameters were as follows: interface temperature: 300 °C; heating block temperature: 250 °C; desolvation line (DL) temperature: 150 °C; spray voltage: 3.00 kV; nebulizer gas (N_2) flow rate: 3 L/min; and heating gas (N_2) and drying gas (N_2) flow rate: 10 L/min. All the experiments were operated in negative ion mode.

Data Processing and Analysis

The average fluorescence intensity of the cell was measured by ImageJ. Statistical analysis was performed using GraphPad Prism 8. Data were presented as the mean ± SD. Student's t-test was used to evaluate the difference between the two groups unless otherwise specified. One-way ANOVA was used to compare the difference between multiple groups, and the Tukey test was used to examine the differences between the two groups.

Data supplementation

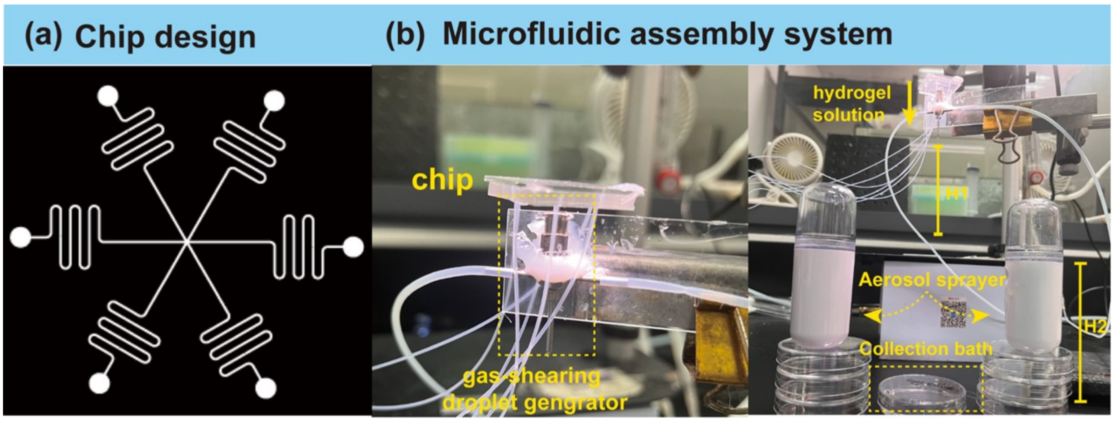

Figure S1. Homemade multicompartmental particle generation microfluidic system. (a) Mask design for the microfluidic chip; (b) Schematic diagram of the microfluidic system setup.

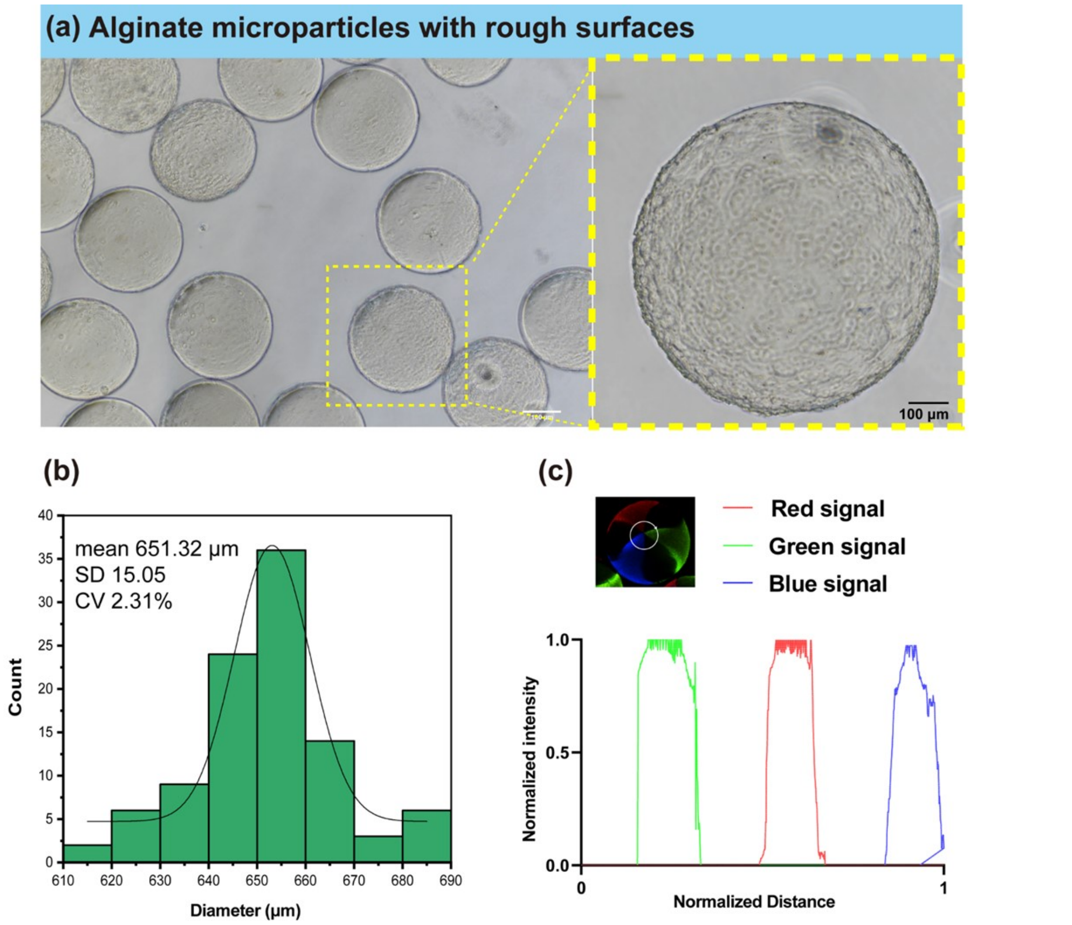

Figure S2. Characterization of multicompartmental hydrogel microspheres with rough surfaces. (a) Morphology of rough-surfaced particles captured under bright-field microscopy; (b) Diameter distribution of rough-surfaced particles; (c) Fluorescence distribution of rough-surfaced particles, scale

bar = 100 μm.

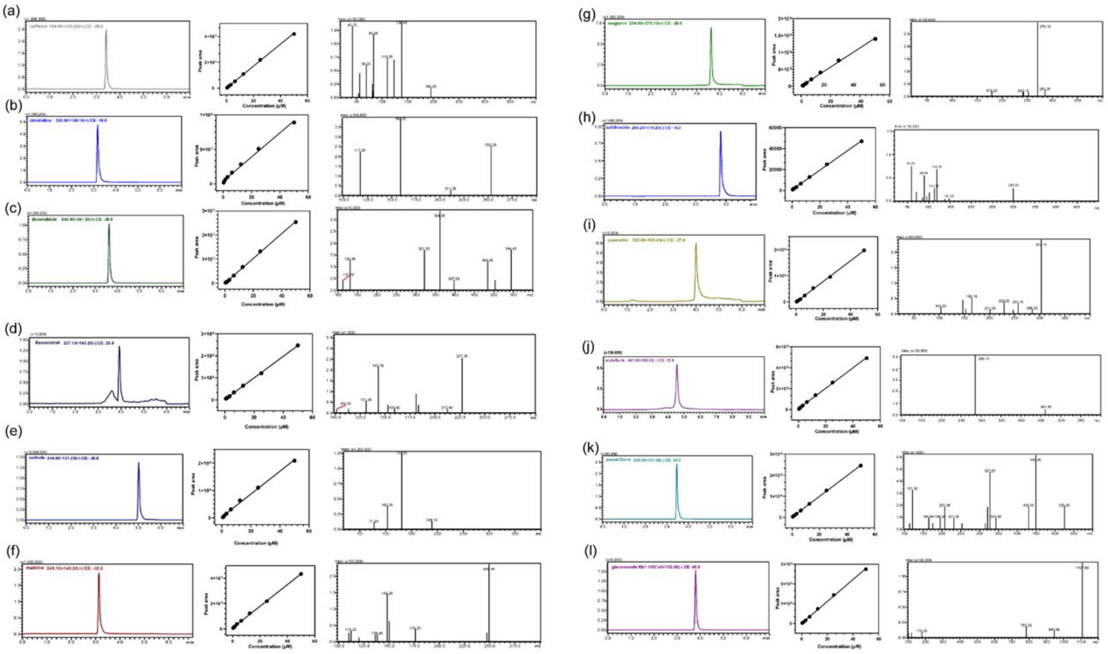

Figure S3. Chromatograms, standard curves, and ion fragmentation spectra of active ingredients with different molecular weights. (a) Caffeine; (b) Cimetidine; (c) Doxorubicin; (d) Resveratrol; (e) Osthole; (f) Matrine; (g) Wogonin; (h) Salidroside; (i) Quercetin; (j) Scutellarin; (k) Paeoniflorin; (l) Ginsenoside Rb1. We obtained standard curves as follow: the peak area (Y) of metabolites increased linearly with the corresponding concentration ratio (X) and the limit of detection were calculated using the following formula: LOD = 3.3 σ/S. The fitting formula is written as follows: Caffeine, Y = 839909 X + 247871 (R2=0.999), as shown in a, LOD is 10.14 nM, Cimetidine, Y = 1749602.35 x + 3851259.91 (R2=0.995), as shown in b, LOD is 18.56 nM, Doxorubicin, Y = 513691X - 20354.3 (R2=0.999), as shown in c, LOD is 10.24 nM, Resveratrol, Y = 4897.52X + 1507.15 (R2=0.999), as shown in d, LOD is 15.56 nM, Osthole, Y = 4171117.67 x + 3308711.22 (R2=0.998), as shown in e, LOD is 15.56 nM, Matrine, Y = 852556X + 825793 (R2=0.999), as shown in f, LOD is 9.42 nM, Wogonin, Y = 2815088.08 x + 1961864.15 (R2=0.998), as shown in g, LOD is 8.25 nM, Salidroside, Y = 936.205X + 676.828 (R2=0.999), as shown in h, LOD is 30.18 nM, Quercetin, Y = 39559.1X - 4450.86 (R2=0.999), as shown in i, LOD is 24.63 nM, Scutellarin, Y = 97873.4X + 83409.3 (R2=0.999), as shown in j, LOD is 5.26 nM, Paeoniflorin, Y = 49079.1X + 21061.4 (R2=0.999), as shown in k, LOD is 4.37 nM, Ginsenoside Rb1, Y = 5559.44X + 58.2439 (R2=0.999), as shown in l, LOD is 5.52 nM.

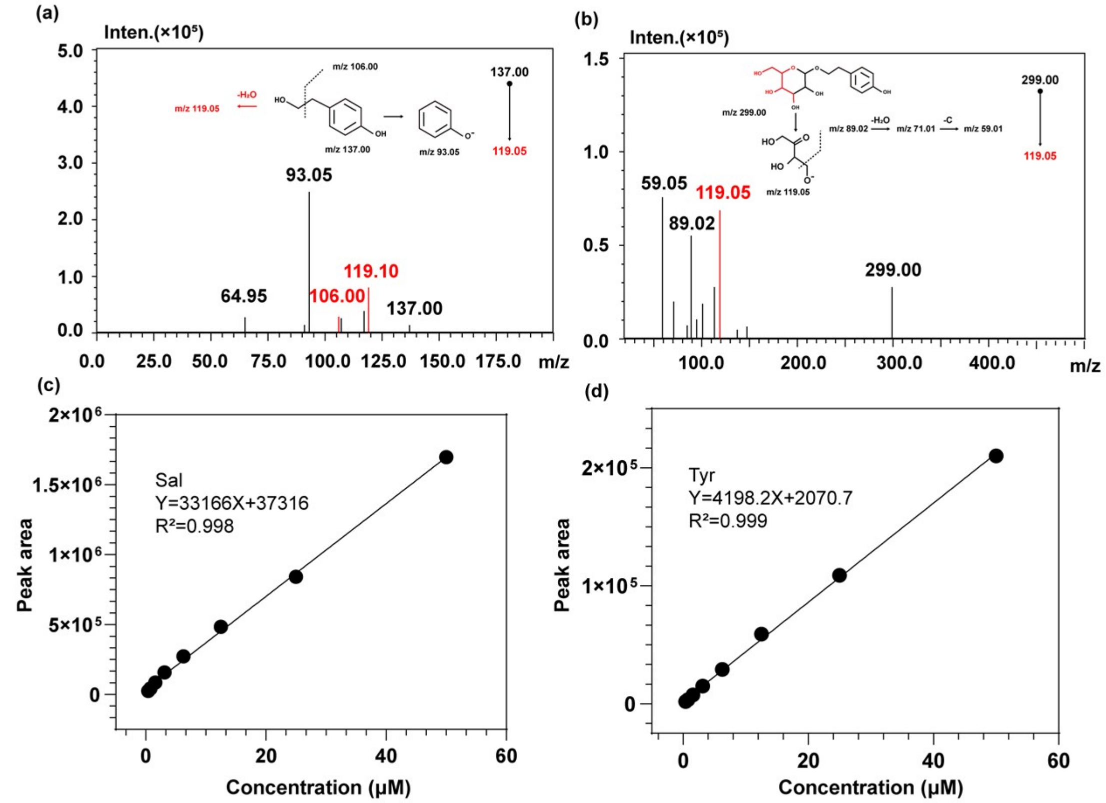

Figure S4. Fragment spectra and calibration curves for salidroside and tyrosol. (a) the fragment spectra of sal; (b) the fragment spectra of tyr; (c) the calibration curves of sal; (d) the calibration curves of tyr.

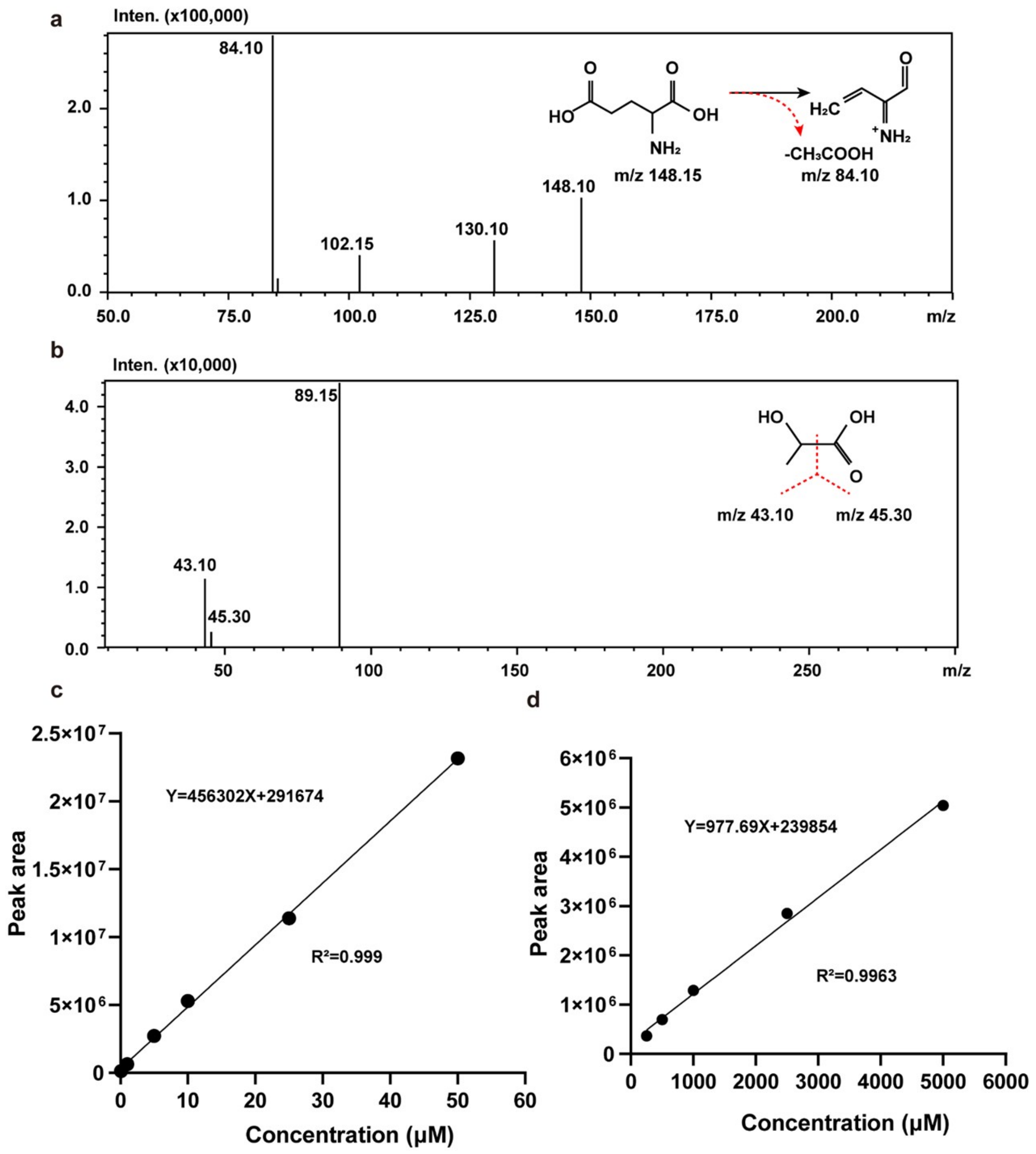

Figure S5. The characteristic fragment structure and standard curves of glutamate and lactate in standard substances. (a) Glutamate, m/z 148.15 → 84.10. (b) Lactate, m/z 89.15 →43.10. Each standard curve was constructed by the mean value of series concentrations, with each concentration detected 3 times. The peak area of metabolites changed linearly with the concentration increasing steadily, and the fitting formula is written as follows: (c) Glutamate, Y = 456302X +291674 (R^2=0.999). (d) Lactate, Y = 977.69X +239854 (R^2=0.996).

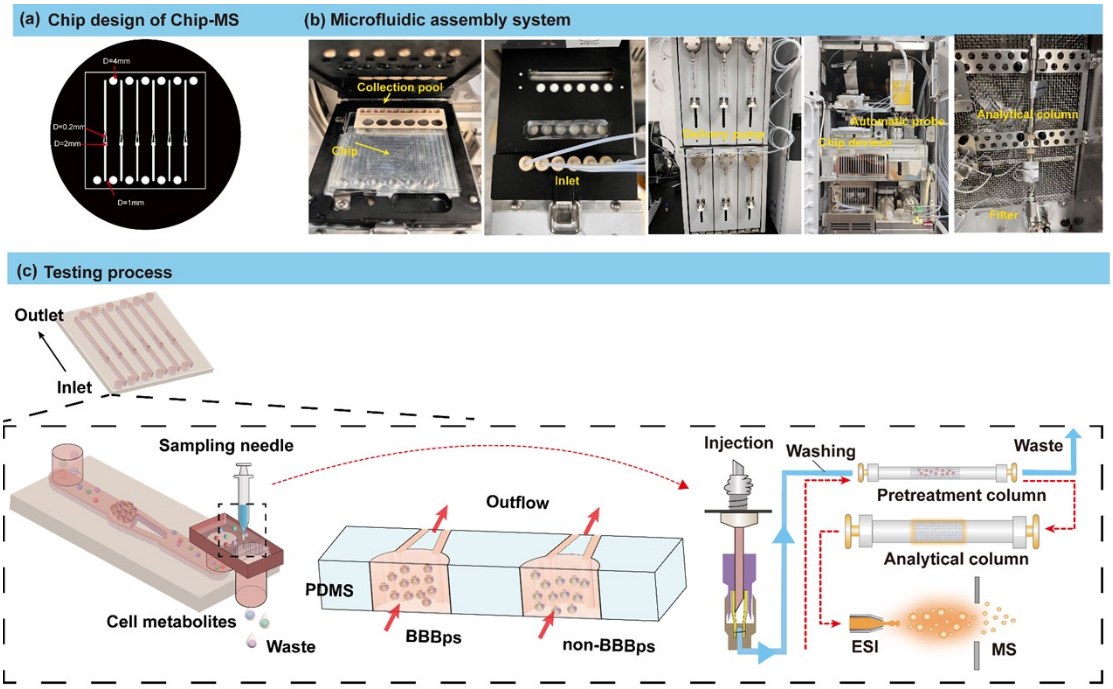

Figure S6. Photographs of the microfluidic chip-mass spectrometry (Chip-MS) device. (a) Dimensions of microfluidic chip. (b) Photograph of microfluidic assembly system. (c) Schematic diagram of the Chip-MS operation process.

Table S1 The MRM mode parameters of 12 target compounds

**Compounds**	t_R	**MRM（*m/z*）**	**CE (eV)**
Caffeine	3.43	194.95>138.20	20
Cimetidine	3.19	253.00>159.15	15
Doxorubicin	3.65	543.90>361.20	28
Resveratrol	3.92	227.10>143.20	25
Osthole	5.02	244.95>131.20	28
Matrine	3.15	249.10>148.30	32
Wogonin	4.60	284.85>270.15	26
Salidroside	3.50	299.20>119.20	14
Quercetin	4.01	302.80>165.20	27
Scutellarin	3.51	461.00>285.00	23
Paeoniflorin	3.48	525.00>121.20	28
Ginsenoside Rb1	3.80	1107.40>783.50	48

Table S2 Comparison of permeability of model drugs (caffeine, cimetidine, and doxorubicin) in BBBps and in situ cerebral perfusion in rodents

Active ingredient	Papp in BBBps(cm/s)	Papp in Transwell (cm/s) [2]	Papp in vivo(cm/s)[5,6]
Caffeine	$1.020(\pm0.001)\times10^{-5}$	$2.42(\pm0.38)\times10^{-5}$	1.65×10^{-4}
Cimetidine	$3.38(\pm0.56)\times10^{-6}$	$4.10(\pm0.78)\times10^{-5}$	1.5×10^{-6}
Doxorubicin	$1.02\ (\pm0.13)\times10^{-6}$	$5.91(\pm3.11)\times10^{-5}$	3.16×10^{-6}

Table S3 Molecular weights and *Papp* data for 12 active ingredients

Active ingredient	Molecular weight (Da)	*Papp (cm/s)*
Caffeine	194.19	$1.020(\pm0.001)\times10^{-5}$
Resveratrol	228.24	$4.78(\pm0.21)\times10^{-6}$
Osthole	244.28	$4.39(\pm0.17)\times10^{-6}$
Matrine	248.36	$4.50(\pm0.53)\times10^{-6}$
Cimetidine	252.34	$3.38(\pm0.56)\times10^{-6}$
Wogonin	284.26	$3.68(\pm0.48)\times10^{-6}$
Salidroside	300.3	$3.94(\pm0.23)\times10^{-6}$
Quercetin	302.23	$3.47(\pm0.46)\times10^{-6}$
Scutellarin	462.4	$2.92(\pm0.06)\times10^{-6}$
Paroniflorin	480.5	$1.26\ (\pm0.25)\times10^{-6}$
Doxorubicin	543.5	$1.02\ (\pm0.13)\times10^{-6}$
Ginsenoside Rb1	1109.3	$6.26\ (\pm2.52)\times10^{-7}$

Table S4 MRM mode parameters of salidroside and tyrosol

**Compounds**	t_R	**MRM（*m/z*）**	**CE (eV)**
Salidroside	3.92	299.00>119.20	14
Tyrosol	4.35	137.00>119.20	17

Table S5 MRM mode parameters of target metabolites

**Compounds**	**MRM（*m/z*）**	**CE (eV)**
Phenylalanine	166.10>103.10	29
Threonine	120.10>74.15	13

Valine	118.00>57.10	30
Glutamate	147.90>84.10	14
Lactate	89.15>43.10	13

Table S6 Gradient elution conditions for 12 active pharmaceutical molecules

Time	Mobile phase A (water, %)	Mobile phase B (acetonitrile, %)
0.5	95	5
1.0	95	5
4.0	5	95
4.5	5	95
5.0	5	95
6.0	95	5

Table S7 Gradient elution conditions for the metabolism of salidroside

Time	Mobile phase A (water, %)	Mobile phase B (acetonitrile, %)
1.50	5	95
2.00	20	80
4.00	20	80
4.50	5	95

Table S8 Gradient elution conditions for amino acids such as threonine and lactate

Time	Mobile phase A (water, %)	Mobile phase B (acetonitrile, %)
0.00	95	5
1.00	95	5
1.50	75	25
2.00	65	35
3.00	5	95
4.00	5	95
4.10	95	5

References

1. Y. Zheng, Z. Wu, Y. Hou, N. Li, Q. Zhang, J.M. Lin. Microfluidic engineering of crater-terrain hydrogel microparticles: toward novel cell carriers. ACS Appl Mater Interfaces, 2023, 15(6):7833-7840.
2. Y. Shi, X. He, H. Wang, J. Dai, J. Fang, Y. He, X. Chen, Z. Hong, Y. Chai. Construction of a novel blood brain barrier-glioma microfluidic chip model: Applications in the evaluation of permeability and anti-glioma activity of traditional Chinese medicine components. Talanta, 2023, 253:123971.
3. Y.I. Wang, H.E. Abaci, M.L. Shuler. Microfluidic blood-brain barrier model provides in vivo-like barrier properties for drug permeability screening. Biotechnology and Bioengineering, 2017, 114 (1): 184-194.
4. N. Xu, H. Lin, S. Lin, W. Zhang, S. Han, H. Nakajima, S. Mao, J.M. Lin. A fluidic isolation-assisted homogeneous-flow-pressure chip-solid phase extraction-mass spectrometry system for online dynamic monitoring of 25-hydroxyvitamin D_3 biotransformation in cells. Anal Chem, 2021, 93(4):2273-2280.
5. L. Shi, M. Zeng, Y. Sun, B.M.J.J.o.B.E. Fu. Quantification of Blood-Brain Barrier solute permeability and brain transport by multiphoton microscopy. Journal of Biomechanical Engineering, 2014, 136 (3): 031005.
6. A. Avdeef. Absorption and drug development: solubility, permeability, and charge state. BMC Medical Research Methodology, 2003, 22-41.
